# The predictors, barriers and facilitators to effective management of acute pain in children by emergency medical services: A systematic mixed studies review

**DOI:** 10.1177/1367493520949427

**Published:** 2020-08-26

**Authors:** Gregory A Whitley, Pippa Hemingway, Graham R Law, Arwel W Jones, Ffion Curtis, Aloysius N Siriwardena

**Affiliations:** 1 Community and Health Research Unit, 4547 University of Lincoln, UK; 2 Faculty of Medicine and Health Sciences, University of Nottingham, UK; 3 Department of Allergy, Immunology and Respiratory Medicine, 2541 Monash University, Australia; 4 Lincoln Institute for Health, 4547 University of Lincoln, UK

**Keywords:** Ambulance, analgesia, children, emergency medical services, pain

## Abstract

We aimed to identify predictors, barriers and facilitators to effective pre-hospital pain management in children. A segregated systematic mixed studies review was performed. We searched from inception to 30-June-2020: MEDLINE, CINAHL Complete, PsycINFO, EMBASE, Web of Science Core Collection and Scopus. Empirical quantitative, qualitative and multi-method studies of children under 18 years, their relatives or emergency medical service staff were eligible. Two authors independently performed screening and selection, quality assessment, data extraction and quantitative synthesis. Three authors performed thematic synthesis. Grading of Recommendations Assessment, Development and Evaluation and Confidence in the Evidence from Reviews of Qualitative Research were used to determine the confidence in cumulative evidence. From 4030 articles screened, 78 were selected for full text review, with eight quantitative and five qualitative studies included. Substantial heterogeneity precluded meta-analysis. Predictors of effective pain management included: ‘child sex (male)’, ‘child age (younger)’, ‘type of pain (traumatic)’ and ‘analgesic administration’. Barriers and facilitators included internal (fear, clinical experience, education and training) and external (relatives and colleagues) influences on the clinician along with child factors (child’s experience of event, pain assessment and management). Confidence in the cumulative evidence was deemed low. Efforts to facilitate analgesic administration should take priority, perhaps utilising the intranasal route. Further research is recommended to explore the experience of the child. Registration: PROSPERO CRD42017058960

## Introduction

Pain is ‘an unpleasant sensory and emotional experience associated with, or resembling that associated with, actual or potential tissue damage’ (International Association for the Study of Pain, 2020). Access to pain management is considered a fundamental human right (Brennan et al., 2019), yet pre-hospital pain management in children is poor (Samuel et al., 2015). This is despite effective pain management being recently identified as a key quality outcome measure for emergency medical (ambulance) services (EMS) (Turner et al., 2019). The management of pain is known to be complex, especially in children, as age, developmental level, cognitive and communication skills, and associated beliefs must be considered (Srouji et al., 2010; Whitley et al., 2019). Without effective pain treatment, children are at risk of adverse consequences including post-traumatic stress disorder (Saxe et al., 2001; Sheridan et al., 2014) and altered pain perception (Taddio et al., 1997; Weisman et al., 1998).

Effective pain management consists of pharmacological and non-pharmacological interventions (Joint Royal Colleges Ambulance Liaison Committee and Association of Ambulance Chief Executives, 2019). Analgesic administration rates for pre-hospital children suffering from acute pain have been low (Lerner et al., 2014; Lord et al., 2016; Whitley and Bath-Hextall, 2017). For example one Australian study (Lord et al., 2016) found that more than half (55%) of children with severe pain (verbal numeric rating scale 8–10) did not receive any analgesics. Non-pharmacological interventions such as slings, splints, bandages and dressings are often missing from datasets or not extracted for analysis and are subsequently cited as a limitation in published research (Lord et al., 2016; Murphy et al., 2017). Other non-pharmacological approaches such as distraction, staying close to relatives and creating a calm environment are rarely documented or extracted for analysis (Pilbery et al., 2019). This lack of data, coupled with the complexity of pre-hospital pain management in children (Whitley et al., 2019), causes uncertainty when attempting to improve quality of care for children suffering from pain.

A comprehensive evidence synthesis is required to provide focus and clarity for future clinical practice interventions and research. This will identify areas of disparity in clinical practice along with known barriers and facilitators. The synthesis of quantitative and qualitative data is necessary to provide unique insights; mixed reviews are less narrow than their single study counterparts and more likely to develop actionable findings to inform policy and practice (Joanna Briggs Institute, 2014).

## Aim

We aimed to identify quantitative predictors of effective pain management along with qualitative barriers and facilitators to the pre-hospital pain management process in children within a systematic mixed studies review.

## Methods

### Study design

**Figure 1.**
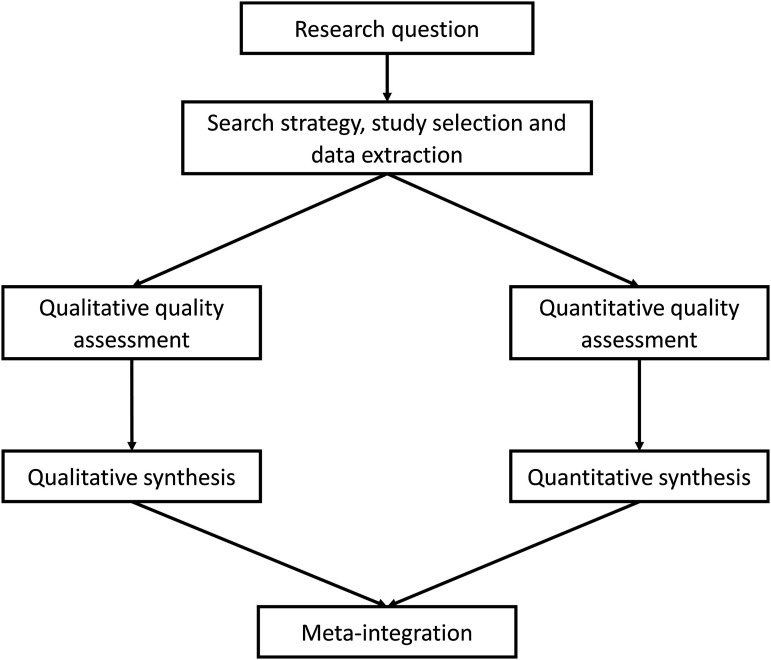
Systematic mixed studies review modified segregated approach. Source: Adapted from Sandelowski et al. (2006) cited in Joanna Briggs Institute (2014).

We used a modified segregated systematic mixed studies approach based on the guidance of Sandelowski et al. (2006), the Joanna Briggs Institute (2014) and the Preferred Reporting Items for Systematic review and Meta-Analysis (PRISMA) guidelines (Liberati et al., 2009). The protocol has previously been published (Whitley et al., 2018). See Figure 1 for the diagram of procedures.

### Eligibility criteria

1. Participants: children (aged <18 years), relatives and emergency medical service staff.
2. Phenomena of interest: studies identifying predictors of ‘effective pain management’ or barriers and facilitators to the pain management process in children suffering from acute pain treated by emergency medical services.
3. Context: international pre-hospital emergency medical services (ambulance, helicopter and other medical rescue services).
4. Types of study: empirical quantitative (interventional, observational and survey) or qualitative designs. Multi-method studies were considered where their component parts could be separated into their respective arm.
5. No language restrictions were placed on the review.

### Search strategy

The search strategy was developed with the assistance of an academic librarian. See Supplementary Material for the search terms used and worked MEDLINE search. The following databases were searched from inception to 30th June 2020:

1. MEDLINE via EBSCOhost
2. CINAHL Complete via EBSCOhost
3. PsycINFO via EBSCOhost
4. EMBASE via Ovid SP
5. Web of Science Core Collection
6. Scopus

Database searching was supplemented with internet searching (e.g. Google Scholar), forward and backward citation tracking from systematic reviews and included studies, and contact with study authors, experts and research groups.

### Study selection

GAW and ANS independently undertook the screening and selection process and resolved any differences in opinion by discussion.

### Data collection

Data extraction was performed by GAW and verified by ANS. There were no disagreements.

### Risk of bias assessment

Quality assessment of included studies was performed in duplicate by GAW and ANS. See protocol for assessment tools used (Whitley et al., 2018). The results were displayed in a risk of bias table (see Supplementary Material). Risk of bias was not used as a reason for exclusion.

### Synthesis

#### Quantitative studies

Where predictors were identified using regression analysis, odds ratios with their 95% confidence intervals were incorporated into the synthesis. Where studies evaluated the effectiveness of analgesics, mean/median pain score reductions were incorporated into the synthesis, along with the percentage of patients achieving effective pain management.

#### Assessment of heterogeneity

Heterogeneity was assessed within STATA version 15 using the ‘metan’ module, incorporating odds ratios and 95% confidence intervals. The *I*^2^ statistic was used to determine heterogeneity. Where substantial heterogeneity was found (*I*^2^ = ≥50%) (Whitley et al., 2018), a narrative analysis was performed.

#### Measurement of treatment effect

The outcome measure was ‘effective pain management’, defined as a pain score reduction of ≥2 out of 10 on the numeric pain rating scale, the Wong–Baker FACES^®^ Scale or the Faces, Legs, Activity, Crying and Consolability Scale (Bailey et al., 2010; Bulloch and Tenenbein, 2002; Myrvik et al., 2013; Tsze et al., 2015; Voepel-Lewis et al., 2011).

#### Subgroup and sensitivity analysis

Sensitivity analyses were not performed due to the low number of studies.

#### Qualitative studies

Thematic synthesis as described by Thomas and Harden (2008) was used to synthesise eligible qualitative studies. This process involves three steps: (1) coding text from the published quotations of eligible studies, (2) developing descriptive themes and (3) generating analytical themes.

#### Meta-integration

After the separate quantitative and qualitative syntheses, meta-integration was performed. Quantitative and qualitative data often address different aspects of a target phenomenon; therefore, they may not be capable of confirming or refuting each other, instead their complementarity can be assessed (Sandelowski et al., 2006). Complementarity is found where data are related to each other linking observations with explanations (Sandelowski et al., 2006) and strengthening the understanding. Where observations and explanations seemed to oppose each other, we used the term ‘conflict’ and recommended further research to explain the disparity. Following the methods of Frantzen and Fetters (2016), this meta-integration was displayed in tabular format to illustrate the complex interrelational connections.

### Meta-bias(es)

No interventional studies were included; therefore, reporting bias could not be assessed. Publication bias was not assessed due to the small number of studies suitable for meta-analysis (*n* = 3).

### Confidence in the cumulative evidence

The Grading of Recommendations Assessment, Development and Evaluation (GRADE) approach (Atkins et al., 2004) was used to assess the quantitative synthesis. The Confidence in the Evidence from Reviews of Qualitative research (CERQual) approach (Lewin et al., 2018) was used to guide the overall assessment of the qualitative synthesis. Overall quality was adjudicated as *High* (further research unlikely to change conclusions), *Moderate* (further research may change conclusions), *Low* (further research likely to change conclusions) or *Very Low* (very uncertain about current conclusions) (Atkins et al., 2004; Lewin et al., 2018; Whitley et al., 2018).

## Results

**Figure 2.**
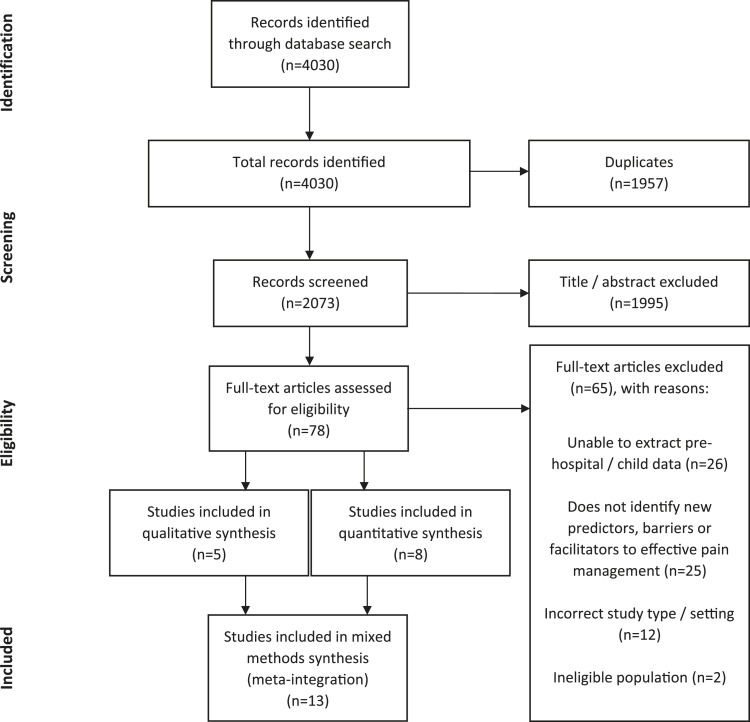
Preferred reporting items for systematic review and meta-analysis flow diagram.

From 4030 articles screened, 78 were selected for full text review, with eight quantitative and five qualitative studies included. See Figure 2 for the PRISMA flow diagram.

A summary of included studies can be seen in Table 1.

**Table 1.** Summary of included studies.

Study	Design	Country	Number of participants	PICo	Primary outcome measure	Key findings
Bendall et al. (2011)	Cross-sectional (Retrospective)	Australia	3312	Participants: Paediatric patients aged 5 to 15 years Phenomena of interest: To compare the effectiveness of intravenous morphine, intranasal fentanyl and inhaled methoxyflurane for managing moderate to severe pain (NPRS ≥5 out of 11) Context: Pre-hospital EMS system	Pain score reduction ≥30%	Intranasal fentanyl and intravenous morphine were equally effective analgesic agents in paediatric patients with moderate to severe acute pain in the out-of-hospital setting. Methoxyflurane was less effective in comparison with both morphine and fentanyl but was an effective analgesic in the majority of children
Jennings et al. (2015)	Cross-sectional (Retrospective)	Australia	15,016	Participants: Children aged <15 years Phenomena of interest: To identify the factors associated with clinically meaningful pain reduction in children Context: Pre-hospital EMS system	Clinically meaningful pain reduction defined as 2 or more out of 11	Patients older than 9 years were less likely and boys were more likely to have a clinically meaningful reduction in pain. Patients with pain classified as musculoskeletal were more likely to achieve a clinically meaningful reduction in pain when compared with other medical causes
Whitley et al. (2020)	Cross-sectional (Retrospective)	United Kingdom	2312	Participants: Children aged <18 years Phenomena of interest: To identify predictors of effective management of acute pain in children Context: Pre-hospital EMS system	Effective pain management, defined as the abolition or reduction of pain by 2 or more out of 10	Predictors included children who were younger, administered analgesics, attended by a paramedic or living in an area of medium or low deprivation
Karlsen et al. (2014)	Cross-sectional (Prospective)	Denmark	903 (63 were less than 18 years of age)	Participants: Adults and children older than 8 years Phenomena of interest: To assess the safety profile and apparent analgesic effect of intranasal fentanyl Context: Pre-hospital EMS system	Occurrence of adverse effects and change in numeric pain score from before fentanyl administration until the last recording before arriving at the hospital	The out-of-hospital administration of intranasal fentanyl in doses of 50 to 100 mcg was safe and appeared effective
Murphy et al. (2017)	Cross-sectional (Prospective)	Ireland	94	Participants: Children aged between 1 and 16 years	Effective reduction in pain, defined as 2 or more out of 11 at 10 minutes following single dose of intranasal fentanyl	INF at a dose of 1.5 μg/kg appeared to be a safe and effective analgesic in the pre-hospital management of acute severe pain in children
				Phenomena of interest: To describe the clinical efficacy and safety of INF when administered by advanced paramedics in the treatment of acute severe pain Context: Pre-hospital EMS system		
Lord et al. (2019)	Interrupted time series analysis (Retrospective)	Australia	9833	Participants: Children aged <15 years Phenomena of interest: To measure the effect that a change to practice guidelines (introduction of INF) for the management of pain in children had on the reduction of pain severity scores in cases receiving analgesics Context: Pre-hospital EMS system	Odds of achieving a 2-point or greater reduction in pain severity score using an 11-point verbal numeric rating scale where any analgesic (morphine, fentanyl or methoxyflurane) was given before and after intervention	Before the intervention, 88.1% (*n* = 3114) of children receiving analgesics had a reduction of pain severity of 2 or more points, with 94.2% (*n* = 5933) achieving this benchmark after intervention (*p* < 0.0001)
Babl et al. (2006)	Case Series (Prospective)	Australia	102	Participants: Children aged 15 months to 17 years Phenomena of interest: To prospectively describe the use pattern of methoxyflurane in children transported to the ED by ambulance Context: Pre-hospital EMS system	Indications for use, verbal numerical pain scores, adverse events and depth of sedation based on paramedic, patient, parent and emergency department staff surveys and review of ambulance care records	Methoxyflurane appeared to be an efficacious analgesic with a low adverse event profile. In young children in particular, it can briefly lead to deep sedation
Johansson et al. (2013)	Case series	Sweden	9 (6 were less than 18 years of age)	Participants: Patients aged 7 to 36 years Phenomena of interest: To describe the use of nasally administered S-ketamine in 9 cases Context: Pre-hospital rural air ambulance	Pain score reduction 5–10 minutes after administration of nasal S-ketamine	VAS-score decreased from a median of 10 to 3. Side effects in these 9 cases were few and non-serious. The effect and safety of this treatment should be further studied
Williams et al. (2012)	Qualitative	United States of America	16	Participants: Paramedics currently in clinical practice Phenomena of interest: To identify and investigate the barriers and enablers perceived by paramedics regarding the administration of analgesics to paediatric emergency medical services patients Context: Pre-hospital EMS system	To identify and investigate the barriers and enablers perceived by paramedics regarding the administration of analgesics to paediatric emergency medical services patients	There was a preference to defer administration of analgesic agents. A number of educational and EMS system changes could be made to address these barriers and increase the frequency of appropriate paediatric pre-hospital analgesia
Murphy et al. (2014)	Qualitative	Ireland	16	Participants: Advanced paramedics with at least 3 years of experience Phenomena of interest: To identify the barriers, as perceived by a national cohort of advanced paramedics, to achieve optimal pre-hospital management of acute pain in children Context: Pre-hospital EMS system	To identify the barriers, as perceived by a national cohort of advanced paramedics, to achieve optimal pre-hospital management of acute pain in children	The pathway to improve care must include an emphasis on improvements in practitioner education and training, offering alternatives to assessing pain in preverbal children, exploring the intranasal route of drug delivery in managing acute severe pain and robustly developed evidence-based guidelines that are practitioner-friendly and patient focused
Gunnvall et al. (2018)	Qualitative	Sweden	8	Participants: Pre-hospital emergency nurses with at least 3 years of experience Phenomena of interest: To examine PENs’ experiences of pain management during pre-hospital care of preverbal children Context: Pre-hospital EMS system	To examine PENs’ experiences of pain management during pre-hospital care of preverbal children, based on PENs’ given mission to alleviate patients’ suffering	A lifeworld perspective with a family-centred approach may support PENs in alleviating pain and suffering in preverbal children. What is required to meet children’s specific needs and security are customised pre-hospital guidelines consisting of both medical and care guidelines; collaboration within a multidisciplinary team and clinical skills and education
Holmstrom et al. (2019)	Qualitative	Sweden	18	Participants: Swedish-speaking pre-hospital emergency nurses with 2 years clinical experience and experience of managing children (0–18 years) in pain Phenomena of interest: To describe nurses’ experiences of pre-hospital care encounters with children in pain and specific related challenges Context: Pre-hospital EMS system	To describe nurses’ experiences of pre-hospital care encounters with children in pain and specific related challenges	Caring for children in pain was stressful for the nurses. The nurses described how they had to shift focus and used different methods to build trust, such as playfulness, making eye contact, attracting curiosity and using the parents to create trust. The also had to adjust to the child regarding dosages and materials
Jepsen et al. (2019)	Qualitative	Sweden	14	Participants: Swedish- or English-speaking parents, whose children had been cared for by the ambulance team Phenomena of interest: To explore the experiences of the caring encounter in the ambulance service among parents to children aged 0–14 years Context: Pre-hospital EMS system	To explore the experiences of the caring encounter in the ambulance service among parents to children aged 0–14 years	There is a need to strengthen the family-centred care in the ambulance service. Not inviting the parents in the care and use of equipment that was non-functioning or not adjustable for the children’s age caused lack of trust and increased the level of stress among the parents

*Note*: PICo: participants, phenomena of interest, context; NPRS: numeric pain rating scale; VAS: visual analogue scale; EMS: emergency medical service; IV: intravenous; IN: intranasal; INF: intranasal fentanyl; ED: emergency department; PEN: pre-hospital emergency nurses.

Risk of bias assessment results can be found in the Supplementary Material. The reporting of observational study design was inconsistent, with Jennings et al. (2015) reporting a cohort study, Bendall et al. (2011) reporting a comparative study, Karlsen et al. (2014) reporting an observational study, Murphy et al. (2017) and Whitley et al. (2020) reporting a cross-sectional study. These five studies were better described as cross-sectional studies and along with Lord et al. (2019) (reporting an interrupted time series analysis) were all assessed using the cross-sectional AXIS tool.

### Quantitative synthesis

Four studies (Bendall et al., 2011; Jennings et al., 2015; Lord et al., 2019; Whitley et al., 2020) used regression analysis to identify predictors of effective pain management. Bendall et al. (2011) used a different outcome measure, where pain reduction ≥30% of the initial pain score was classified as ‘effective pain management’, so an exception was made to include this study. Jennings et al. (2015) and Lord et al. (2019) used the same base dataset; therefore, the predictor ‘child sex’ was excluded for Lord et al. (2019). Substantial heterogeneity (*I*^2^ of 60.5%) was found for the ‘child sex’ predictor (see Supplementary Material); therefore, a narrative synthesis was performed. See Table 2 for identified predictors.

**Table 2.** Factors predicting effective pain management.

Predictor (odds of achieving effective* pain reduction)	Study			
	Bendall et al. (2011) AOR (95% CI)	Jennings et al. (2015)** AOR (95% CI)	Lord et al. (2019)** AOR (95% CI)	Whitley et al. (2020) AOR (95% CI)
Child sex				
Male	1.42 (1.19–1.71)	1.1 (1.0–1.3)		1.17 (0.98–1.39)
Child age, years				
5–9 (compared to 10–15)	1.33 (1.00–1.75)			
5–9 (compared to 0–4)		0.7 (0.6–0.95)		
10–14 (compared to 0–4)		0.5 (0.4–0.6)		
>9 (compared to <3)			0.49 (0.23–1.06)	
0–5 (compared to 12–17)				1.53 (1.18–1.97)
6–11 (compared to 12–17)				1.49 (1.21–1.82)
Type of pain				
Abdominal pain/problems (compared to trauma)	0.69 (0.50–0.96)^a^			
Musculoskeletal (compared to medical)		1.7 (1.5–1.9)		
Burns (compared to medical)		1.6 (1.1–2.5)		
Trauma (other) (compared to medical)		1.4 (1.1–1.9)		
Cardiac (compared to musculoskeletal)			0.22 (0.08–0.60)	
Trauma (compared to medical)				1.18 (0.97–1.43)
Initial pain score				
Moderate (4–7/10) (compared to 3/10)		3.9 (3.3–4.6)		
Severe (8–10/10) (compared to 3/10)		7.5 (6.2–9.0)		
Analgesic agent				
Methoxyflurane (compared to IV morphine)	0.52 (0.36–0.74)			
Methoxyflurane (compared to IN fentanyl)	0.43 (0.29–0.62)			
Methoxyflurane (compared to no analgesia)		5.3 (4.8–5.9)		
Fentanyl (IN & IV) (compared to no analgesia)		2.8 (2.3–3.3)		
Morphine (IV) (compared to no analgesia)		2.8 (2.2–3.6)		
Any analgesic (compared to no analgesic)		6.6 (5.9–7.3)		
Analgesic administered (compared to no analgesic)				2.26 (1.87–2.73)
Index of multiple deprivation^b^				
Low deprivation (compared to high deprivation)				1.37 (1.04–1.80)
Medium deprivation (compared to high deprivation)				1.41 (1.11–1.79)
Clinician rank				
Paramedic crew (compared to non-paramedic crew)				1.46 (1.19–1.79)
Implementation of IN fentanyl				
After implementation of IN fentanyl (compared to before implementation)			2.33 (1.71–3.17)	
Trend after intervention on IN fentanyl (compared to before implementation)			0.97 (0.95–1.0)	

*Note*: AOR: adjusted odds ratio; CI: confidence interval; IN: intranasal; IV: intravenous.

*Bendall et al. (2011) (reduction ≥30%), Jennings et al. (2015), Lord et al. (2019) and Whitley et al. (2020) (reduction ≥2/10).

**Jennings et al. (2015) and Lord et al. (2019) used the same base dataset; therefore, the predictor ‘child sex’ was excluded for Lord et al. (2019).

^a^Unadjusted odds ratio.

^b^Index of multiple deprivation data from UK ministry of housing, communities and local government 2015 (deciles used and categorised as 1–3 (low), 4–7 (medium) and 8–10 (high).

Table 2 shows that child sex (male), child age (younger), type of pain (traumatic), initial pain score (moderate/severe), analgesic administration, level of deprivation (low/medium), the presence of a paramedic and implementation of intranasal fentanyl were all predictors of effective pain management. However, the trend after implementation of intranasal fentanyl, which demonstrated a downward slope (gradual decline in proportion of patients achieving a 2+ point reduction in pain), was not associated with effective pain management. Jennings et al. (2015) used ‘initial pain score’ to calculate the dependent variable (effective pain management) and as an independent variable; therefore, their findings may be influenced by mathematical coupling (Archie, 1981).

Four studies evaluated the effectiveness of specific analgesics (Babl et al., 2006; Johansson et al., 2013; Karlsen et al., 2014; Murphy et al., 2017). It was found that intranasal fentanyl with and without additional analgesics, methoxyflurane with additional analgesics and nasal S-ketamine with other analgesics, were all predictors of effective pain management (see Supplementary Material). For the purpose of this analysis, we grouped these predictors along with analgesic agents seen in Table 2 as ‘analgesic administration’.

None of the cross-sectional studies justified the sample size used. There were some concerns regarding the appropriateness of the target population, as Karlsen et al. (2014) excluded children under 9 years of age and Murphy et al. (2017) recruited advanced paramedics; the findings of these studies may not be representative of the wider EMS clinician or patient population. See Supplementary Material for the risk of bias assessments for cross-sectional and case series studies.

### Qualitative synthesis

Five studies were included in the qualitative synthesis, interviewing paramedics (Williams et al., 2012), advanced paramedics (Murphy et al., 2014), pre-hospital emergency nurses (Gunnvall et al., 2018; Holmström et al., 2019) and parents of children (Jepsen et al., 2019).

**Figure 3.**
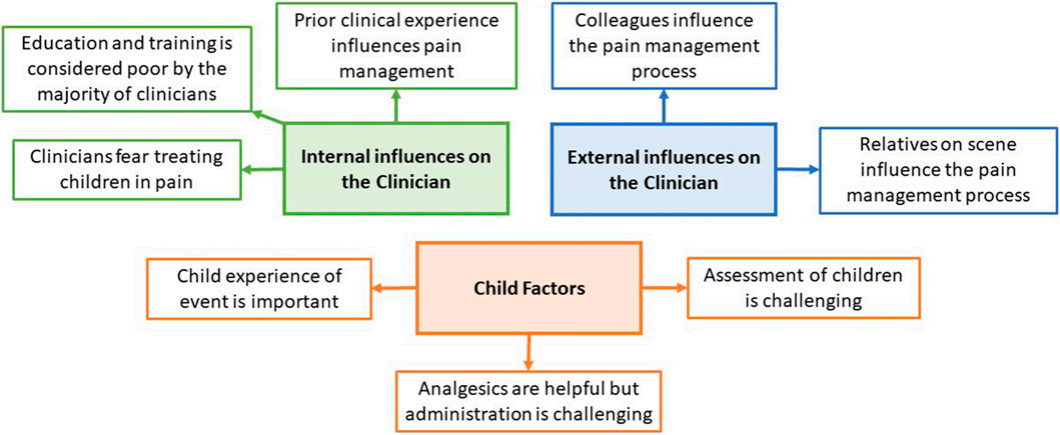
Thematic synthesis: analytical and descriptive themes.

Thematic synthesis resulted in the generation of three analytical themes: internal influences on the clinician, external influences on the clinician and child factors. These analytical themes were generated from eight descriptive themes (See Figure 3) which in turn were linked to 36 initial codes (see Supplementary Material). For a complete list of known barriers and facilitators, see initial codes within Supplementary Material.

#### Internal influences on the clinician

A major theme arising from the evidence was the element of fear within the clinician (see quotations 17–21 of thematic synthesis in Supplementary Material). In addition to fearing the side effects of strong analgesics, clinicians feared making mistakes due to insufficient experience or insecurity (Gunnvall et al., 2018) and feared potential punishment for such errors (Williams et al., 2012), all of which necessitate increased vigilance and extra supervision of drug doses (Holmström et al., 2019).

Clinicians felt unprepared, as many deemed their education and training inadequate (see quotations 3–6 of thematic synthesis in Supplementary Material). Clinicians received very little time on placement within paediatric emergency departments, and formal clinical assessments on children were restricted due to fear of further distressing the child (Murphy et al., 2014). Some clinicians received no specific training and education for children in the pre-hospital setting (Gunnvall et al., 2018). Education was sparse for child pain assessment tools (Holmström et al., 2019), and some clinicians even recalled being taught to look for reasons not to give morphine during their education and training (Williams et al., 2012). A facilitator was identified by Murphy et al. (2014) in the form of e-learning, which could be used to overcome some of these educational barriers.

Prior clinical experience was found to influence the pain management process, with many clinicians suffering from a lack of exposure (see quotations 28–31 of thematic synthesis in Supplementary Material). Clinicians experienced higher rates of stress when attending children, likely exaggerated by the lack of clinical experience and low rates of exposure (Holmström et al., 2019; Williams et al., 2012). Prior clinical experience could be beneficial, allowing clinicians to recognise painful presentations faster, speeding up the assessment process (Williams et al., 2012). However, experience could facilitate clinicians to adopt social and cultural norms where traumatic pain is treated more readily than medical pain (Murphy et al., 2014).

#### External influences on the clinician

The level of support from colleagues and relatives on scene varied among clinicians. Many felt that colleagues were unsupportive (see quotations 35–37, 41 and 44–45 of thematic synthesis in Supplementary Material), yet others found colleagues supportive (see quotations 38–40, 42 and 43 of thematic synthesis in Supplementary Material). Some clinicians wanted to administer analgesics when a general practitioner had withheld them (Murphy et al., 2014), some were concerned about what the hospital staff would say with regard to their treatment (Murphy et al., 2014; Williams et al., 2012), some were inspired by a mentor to be more liberal with their management of pain (Williams et al., 2012) and others stated that their crewmate was helpful to either manage ‘hysterical’ parents (Holmström et al., 2019) or switch to attending the child (Gunnvall et al., 2018).

There appeared to be disagreement with perceived support of relatives on scene, with some describing relatives as helpful (see quotations 48–53 of thematic synthesis in Supplementary Material). From the parent’s perspective, they found that being involved helped the assessment of their child (Jepsen et al., 2019). Other clinicians however felt that relatives hindered the pain management process (see quotations 54–56 of thematic synthesis in Supplementary Material). Some clinicians stated that parents can be ‘hysterical’ (Holmström et al., 2019) and confrontational (Williams et al., 2012) which can inhibit the clinician’s ability to effectively manage pain. Jepsen et al. (2019) explored the parent’s perspective of the care encounter with the ambulance service and highlighted the importance of a family-centred approach that included the child and parents. Therefore, clinicians should prioritise calming and relaxing the parents as this will likely be reflected in the child (Gunnvall et al., 2018).

#### Child factors

Clinicians felt the experience of the child was an important consideration (see quotation 57 of thematic synthesis in Supplementary Material). There was a strong appreciation for the holistic approach, particularly from Swedish clinicians who preferred to treat children in their own home (Gunnvall et al., 2018), include them in the decision making process (Gunnvall et al., 2018) and prioritised the development of trust with the child (Gunnvall et al., 2018; Holmström et al., 2019). Clinicians also considered the risk versus benefit of gaining intravenous access, acknowledging the additional pain it would cause (Holmström et al., 2019; Murphy et al., 2014; Williams et al., 2012).

It was clear that the administration of analgesics was challenging, particularly in younger children (see quotations 73–75 of thematic synthesis in Supplementary Material). There were concerns about the difficulty in gaining intravenous access (Holmström et al., 2019; Murphy et al., 2014), difficulty administering inhaled analgesics (Murphy et al., 2014) and determining a child’s weight (Williams et al., 2012). Many clinicians hinted that the intranasal route was a promising alternative to overcome the current barriers of analgesic administration (Holmström et al., 2019; Murphy et al., 2014).

Clinicians stated that the assessment of pain was challenging, more so in younger children (see quotations 83–87 and 89–91 of thematic synthesis in Supplementary Material). Some clinicians stated that younger children were more difficult to assess, in part due to communication difficulty (Gunnvall et al., 2018; Murphy et al., 2014), whilst others stated that older children were more difficult to assess (Holmström et al., 2019). Clinicians also relied on physiological signs to determine the child’s severity of pain, such as level of play and curiosity (Gunnvall et al., 2018) along with signs such as tachycardia (Williams et al., 2012).

### Meta-integration

Only predictors of effective pain management identified from more than one source were incorporated into the meta-integration. Of the four included predictors, two were complemented, one conflicted and one unexplained by the qualitative synthesis (see Table 3).

**Table 3.** Meta-integration.

Quantitative synthesis (predictors)		Qualitative synthesis (barriers and facilitators)	Integration (complement/conflict/unexplained)
*Child sex*		NULL	The predictor of effective pain management ‘child sex (male)’ was **unexplained** because there was no qualitative data exploring the association between child sex and effective pain management. Further research is required.
Male children are more likely to achieve effective pain management than female children			
*Child age*	Initial codes	Younger children are more difficult to assess	The predictor of effective pain management ‘child age (younger)’ was **conflicted** by the qualitative synthesis which found that younger children are more difficult to assess, cannulate and administer inhaled analgesics.
Younger children are more likely to achieve effective pain management than older children			
		IV access is difficult, especially in younger children	
		Inhaled analgesics are difficult to administer to younger children	
	Descriptive themes	Assessment of children is challenging	
		Analgesics are helpful but administration is challenging	
	Analytical theme	Child factors	
*Type of pain*	Initial code	Decision-making; trauma is treated more readily than medical pain	The predictor of effective pain management ‘type of pain (traumatic)’ was **complemented** by the qualitative synthesis which found clinicians, as part of the decision-making process, treat traumatically injured children more readily than those with medical pain.
Traumatically injured children are more likely to achieve effective pain management than those with medical aetiologies			
	Descriptive theme	Prior clinical experience influences pain management	
	Analytical theme	Internal influences on the clinician	
*Analgesic administration*	Initial codes	Analgesia improves child anxiety and compliance	The predictor of effective pain management ‘analgesic administration’ was **complemented** by the qualitative synthesis which mostly explained the decision-making process rather than its effectiveness. The synthesis found that analgesics are helpful but restrictive clinical guidelines hinder effective pain management.
Children who receive analgesics are more likely to achieve effective pain management than those who do not			
		Restrictive clinical guidelines inhibit effective pain management	
	Descriptive themes	Analgesics are helpful but administration is challenging	
		Education and training is considered poor by the majority of clinicians	
	Analytical themes	Child factors	
		Internal influences on the clinician	

Complement: data are related to each other linking observations with explanations; Conflict: observations and explanations seem to oppose each other; NULL: no data; IV: intravenous.

### Confidence in the cumulative evidence

We used GRADE (Atkins et al., 2004) and CERQual (Lewin et al., 2018) guidelines to assess confidence in the cumulative evidence which was deemed low. Further research is likely to have an important impact on our confidence in the estimate of effect and is likely to change the estimate. This was due to the low-quality design (observational) of the studies informing the quantitative findings and the minor concerns regarding the methodological limitations and relevance of the studies informing the qualitative findings. See Supplementary Material for the GRADE assessment, CERQual evidence profile and CERQual summary of qualitative findings.

## Discussion

### Main findings

Predictors of effective pain management included ‘child sex (male)’, ‘child age (younger)’, ‘type of pain (traumatic)’ and ‘analgesic administration’. Barriers and facilitators included internal (fear, clinical experience, education and training) and external (relatives, colleagues) influences on the clinician along with child factors (child’s experience of event, pain assessment and management). Meta-integration of quantitative and qualitative data showed links between observation and explanation (complementarity) for the predictors ‘analgesic administration’ and ‘type of pain (traumatic)’, opposing links (conflict) for the predictor ‘child age (younger)’ and no links (unexplained) for the predictor ‘child sex (male)’.

Clinicians had strong concerns about analgesics, emphasising barriers such as wide dosing regimes, associated risks and concerns about their strength. These concerns necessitate more decision-making by the clinician; however, this may leave clinicians feeling more vulnerable to criticism by peers and senior authorities within the EMS system; these are known barriers identified within this review.

Clinicians expressed a preference for the intranasal route of administration of analgesics (Holmström et al., 2019; Murphy et al., 2014), resulting in changes to clinical practice as evidenced by Murphy et al. (2017) and Lord et al. (2019). Administering via the intranasal route overcomes some of the barriers to pain management such as difficulty cannulating and difficulty administering inhaled analgesics to younger children. Considering the strong links between quantitative and qualitative data, efforts to facilitate the administration of analgesics should be taken as a priority in clinical practice and future research.

The predictor ‘child age’ demonstrated conflict during meta-integration, as quantitative data suggested younger children are more likely to achieve effective pain management, yet qualitative data suggested barriers such as younger children are more difficult to assess and treat. Samuel et al. (2015) suggested that smaller children may be less likely to receive analgesics because of the difficulty in assessing pain. Accurate pain assessment in children requires the appropriate use of validated tools such as the Wong–Baker FACES^®^ Scale which has been validated in the acute setting (Garra et al., 2010). There is potential for inaccuracies in the measurement of pain or inappropriate use of pain scales, potentially overestimating the effect of pain management strategies in younger children who can less clearly verbalise their experience. Clinicians should ensure that they are using pain assessment scales as validated, for example the Wong-Baker FACES^®^ Scale should be used as a self-assessment tool for the child to use rather than for the clinician to use as an objective measure.

Meta-integration could not be performed for the predictor ‘child sex (male)’ due to the lack of qualitative data. The disparity in perceived pain between the two sexes exists from an early age (Guinsburg et al., 2000) and continues through to adolescence (Keogh and Eccleston, 2006). However, explanations for this difference are sparse. In children who are able to speak, these data could be explained by male children acting ‘tough’ or being ‘brave’, playing down the pain and more readily reporting pain relief post-intervention than their female counterparts. Equally, unconscious gender bias on the part of the clinician when administering treatments could influence this disparity. Further qualitative research is required to assess these theories.

There were strong links between the identified predictor ‘type of pain (traumatic)’ and the qualitative findings of the thematic synthesis, which showed that trauma is treated more readily than medical pain. This preferential treatment of children with traumatic injuries should be addressed, as children with medical causes of pain are more likely to suffer unnecessarily.

### Strengths and limitations

The strength of this review lies in its mixed approach, supplying context and enriching the quantitative findings with qualitative data. This has produced novel findings not previously identified, such as the conflict surrounding ‘child age (younger)’. To our knowledge, this is the first systematic review focusing on predictors, barriers and facilitators to effective management of acute pain in children within the pre-hospital setting.

These findings are generalisable because studies of urban, rural and mixed EMS systems from Europe, Australasia and North America were incorporated into this review. Also, the qualitative data were comprised from a broad range of clinicians, including paramedics, advanced paramedics and pre-hospital emergency nurses.

A limitation was the confidence in the cumulative evidence being deemed low; further research is likely to change the conclusions. This review should be updated in light of new evidence.

Analgesic administration may not predict effective pain management in children suffering from mild pain, as the studies contributing to the predictor ‘analgesic administration’ were mostly in the context of moderate to severe pain (Bendall et al., 2011; Jennings et al., 2015; Karlsen et al., 2014; Lord et al., 2019; Murphy et al., 2017).

The voice of the child was not heard in this review; however, we feel that the diverse group of clinicians along with the parents’ perspective provided a balanced account encompassing a wide variety of barriers and facilitators.

#### Implications for clinical practice

Efforts to improve clinical practice should focus on the following:

1. Explore ways to facilitate analgesic administration; specifically, the intranasal route of administration should be explored within pre-hospital EMS systems. Barriers and facilitators identified within the thematic synthesis of this review should be addressed, perhaps through educational interventions, such as utilising e-learning packages and improving clinical support.
2. Address the culture of managing traumatic pain more readily than pain arising from medical conditions; education and training centres should emphasise the importance of effective pain management for both traumatic and medical sources of pain.

#### Implications for future research

Future research should explore the conflict surrounding the predictor ‘child age (younger)’ as the evidence here appears to conflict. The predictor ‘child sex (male)’ should also be explored, as this remains unexplained. The perceptions and experience of the child should also be explored in research that elicits the child’s voice, as this was clearly lacking from the evidence base.

## Conclusion

Predictors of effective pain management were identified, along with perceived barriers and facilitators. Further studies investigating methods to facilitate analgesic administration (e.g. the intranasal route) are a priority. Further research is recommended to explore the conflict around ‘child age (younger)’, the unexplained data around ‘child sex (male)’ and the experience of the child.

## Supplemental Material

sj-pdf-1-chc-10.1177_1367493520949427 – Supplemental Material for The predictors, barriers and facilitators to effective management of acute pain in children by emergency medical services: A systematic mixed studies review Click here for additional data file. Supplemental Material, sj-pdf-1-chc-10.1177_1367493520949427 for The predictors, barriers and facilitators to effective management of acute pain in children by emergency medical services: A systematic mixed studies review by Gregory A Whitley, Pippa Hemingway, Graham R Law, Arwel W Jones, Ffion Curtis and Aloysius N Siriwardena in Journal of Child Health Care
