# Supplementary material for: The predictors, barriers and facilitators to effective management of acute pain in children by emergency medical services: A systematic mixed studies review
Source: J Child Health Care. 2020 Aug 26;25(3):481–503. doi: 10.1177/1367493520949427 (PMC8422593; doi:10.1177/1367493520949427)
Supplement: sj-pdf-1-chc-10.1177_1367493520949427 – Supplemental Material for The predictors, barriers and facilitators to effective management of acute pain in children by emergency medical services: A systematic mixed studies review [file sj-pdf-1-chc-10.1177_1367493520949427.pdf]

Supplementary File

Contents

Search strategy 2

Worked search, MEDLNE via EBSCOhost 3

Risk of bias assessments 4

Heterogeneity assessment of the child sex predictor 8

Predictive analgesics 10

Thematic synthesis 11

GRADE assessment 19

CERQual evidence profile 23

CERQual summary of qualitative findings 25

References 26

Search strategy

<table>
<tr><td></td><td colspan="6">Database</td></tr>
<tr><td></td><td>MEDLINE</td><td>CINAHL complete</td><td>PsycINFO</td><td>EMBASE</td><td>Scopus</td><td>Web of Science Core Collection</td></tr>
<tr><td>S1</td><td>Infant* OR Child* OR Pediatric* OR Paediatric* OR Adolescen* OR (MH “Pediatrics”) OR (MH “Adolescent”)</td><td>Infant* OR Child* OR Pediatric* OR Paediatric* OR Adolescen* OR (MH “Pediatrics”) OR (MH “Adolsecence”)</td><td>Infant* OR Child* OR Pediatric* OR Paediatric* OR Adolescen* OR DE “Pediatrics”</td><td>Infant* OR Child* OR Pediatric* OR Paediatric* OR Adolescen* OR Pediatrics/ OR adolescent/ OR child/</td><td rowspan="4">TITLE-ABS-KEY ((infant* OR child* OR pediatric* OR paediatric* OR adolescen*) AND (ambulance* OR "Emergency Medical Service*" OR prehospital OR pre-hospital OR "Out of hospital" OR paramedic*) AND (pain OR analgesi*))</td><td rowspan="4">TS=((Infant* OR Child* OR Pediatric* OR Paediatric* OR Adolescen*) AND (Ambulance* OR "Emergency Medical Service*" OR Prehospital OR Pre-hospital OR "Out of hospital" OR Paramedic*) AND (Pain OR Analgesi*))</td></tr>
<tr><td>S2</td><td>Ambulance* OR "Emergency Medical Service*" OR Prehospital OR Pre-hospital OR "Out of hospital" OR Paramedic* OR (MH “Emergency Medical Services”) OR (MH “Ambulances”)</td><td>Ambulance* OR "Emergency Medical Service*" OR Prehospital OR Pre-hospital OR "Out of hospital" OR Paramedic* OR (MH “Emergency Medical Services”) OR (MH “Ambulances”)</td><td>Ambulance* OR "Emergency Medical Service*" OR Prehospital OR Pre-hospital OR "Out of hospital" OR Paramedic* OR DE “Emergency Services”</td><td>Ambulance* OR "Emergency Medical Service*" OR Prehospital OR Pre-hospital OR "Out of hospital" OR Paramedic* OR ambulance/</td></tr>
<tr><td>S3</td><td>Pain OR Analgesi* OR (MH “Acute Pain”) OR (MH “Pain Management”)</td><td>Pain OR Analgesi* OR (MH “Pain”) OR (MH “Pain Management”)</td><td>Pain OR Analgesi* OR DE “Pain”</td><td>Pain OR Analgesi* OR Pain/ OR analgesia/</td></tr>
<tr><td>S4</td><td>S1 AND S2 AND S3</td><td>S1 AND S2 AND S3</td><td>S1 AND S2 AND S3</td><td>S1 AND S2 AND S3</td></tr>
</table>

Worked search, MEDLNE via EBSCOhost

EBSCOhost

Tuesday, June 30, 2020 6:43:29 AM

#	Query	Limiters/Expanders	Last Run Via	Results
S4	S1 AND S2 AND S3	Expanders - Apply equivalent subjects<br>Search modes - Boolean/Phrase	Interface - EBSCOhost Research Databases<br>Search Screen - Basic Search<br>Database - MEDLINE	1,022
S3	Pain OR Analgesi* OR (MH "Acute Pain") OR (MH "Pain Management")	Expanders - Apply equivalent subjects<br>Search modes - Boolean/Phrase	Interface - EBSCOhost Research Databases<br>Search Screen - Basic Search<br>Database - MEDLINE	833,050
S2	Ambulance* OR "Emergency Medical Service*" OR Prehospital OR Pre-hospital OR "Out of hospital" OR Paramedic* OR (MH "Emergency Medical Services") OR (MH "Ambulances")	Expanders - Apply equivalent subjects<br>Search modes - Boolean/Phrase	Interface - EBSCOhost Research Databases<br>Search Screen - Basic Search<br>Database - MEDLINE	82,284
S1	Infant* OR Child* OR Pediatric* OR Paediatric* OR Adolescen* OR (MH "Pediatrics") OR (MH "Adolescent")	Expanders - Apply equivalent subjects<br>Search modes - Boolean/Phrase	Interface - EBSCOhost Research Databases<br>Search Screen - Basic Search<br>Database - MEDLINE	4,441,697

Risk of bias assessments

Cross-sectional study quality / risk of bias assessment

Question	Study					
	**Bendall et al (2011)** [1]	**Jennings et al (2015)** [2]	**Karlsen et al (2014)** [3]	**Lord et al (2019)** [4]	**Murphy et al (2017)** [5]	**Whitley et al (2020) [6]**
1. Were the aims/objectives of the study clear?						
2. Was the study design appropriate for the stated aim(s)?						
3. Was the sample size justified?						
4. Was the target/reference population clearly defined? (Is it clear who the research was about?)						
5. Was the sample frame taken from an appropriate population base so that it closely represented the target/reference population under investigation?						
6. Was the selection process likely to select subjects/participants that were representative of the target/reference population under investigation?						
8. Were the risk factor and outcome variables measured appropriate to the aims of the study?						
9. Were the risk factor and outcome variables measured correctly using instruments/measurements that had been trialled, piloted or published previously?						
10. Is it clear what was used to determined statistical significance and/or precision estimates? (eg, p values, CIs)						
11. Were the methods (including statistical methods) sufficiently described to enable them to be repeated?						
12. Were the basic data adequately described?						

15. Were the results internally consistent?	Yes	Yes	Yes	Yes	Yes	Yes
16. Were the results for the analyses described in the methods, presented?	Yes	Yes	Yes	Yes	Yes	Yes
17. Were the authors' discussions and conclusions justified by the results?	Yes	Yes	Yes	Yes	Yes	Yes
18. Were the limitations of the study discussed?	Yes	Yes	Yes	Yes	Yes	Yes
19. Were there any funding sources or conflicts of interest that may affect the authors' interpretation of the results?	Unclear	Unclear	Yes	Unclear	Yes	Yes
20. Was ethical approval or consent of participants attained?	Yes	Yes	Yes	Yes	Yes	Yes

AXIS tool used [7]

Key:

**Yes**
**Unclear**
**No**
**N/A**

Case series study quality / risk of bias assessment

Question	Study	
	**Babl et al (2006)** [8]	**Johansson et al (2013)** [9]
1. Were there clear criteria for inclusion in the case series?		
2. Was the condition measured in a standard, reliable way for all participants included in the case series?		
3. Were valid methods used for identification of the condition for all participants included in the case series?		
4. Did the case series have consecutive inclusion of participants?		
5. Did the case series have complete inclusion of participants?		
6. Was there clear reporting of the demographics of the participants in the study?		
7. Was there clear reporting of clinical information of the participants?		
8. Were the outcomes or follow up results of cases clearly reported?		
9. Was there clear reporting of the presenting site(s)/clinic(s) demographic information?		
10. Was statistical analysis appropriate?		

Joanna Briggs Institute tool used [10]

Key:

**Yes**
**Unclear**
**No**

Qualitative study quality / risk of bias assessment

Question	Study				
	**Jepsen et al (2019)** [11]	**Holmstrom et al (2019)** [12]	**Gunnvall et al (2018)** [13]	**Murphy et al (2014)** [14]	**Williams et al (2012)** [15]
1. Was there a clear statement of the aims of the research?	Yes	Yes	Yes	Yes	Yes
2. Is a qualitative methodology appropriate?	Yes	Yes	Yes	Yes	Yes
3. Was the research design appropriate to address the aims of the research?	Yes	Yes	Yes	Yes	Yes
4. Was the recruitment strategy appropriate to the aims of the research?	Yes	Yes	Yes	Unclear	Yes
5. Was the data collected in a way that addressed the research issue?	Yes	Yes	Yes	Yes	Yes
6. Has the relationship between researcher and participants been adequately considered?	Yes	Yes	Yes	No	Yes
7. Have ethical issues been taken into consideration?	Yes	Yes	Yes	Yes	Yes
8. Was the data analysis sufficiently rigorous?	Yes	Yes	Yes	Unclear	Unclear
9. Is there a clear statement of findings?	Yes	Yes	Yes	Yes	Yes

Qualitative CASP tool used [16]

Key:

**Yes**
**Unclear**
**No**

Heterogeneity assessment of the child sex predictor

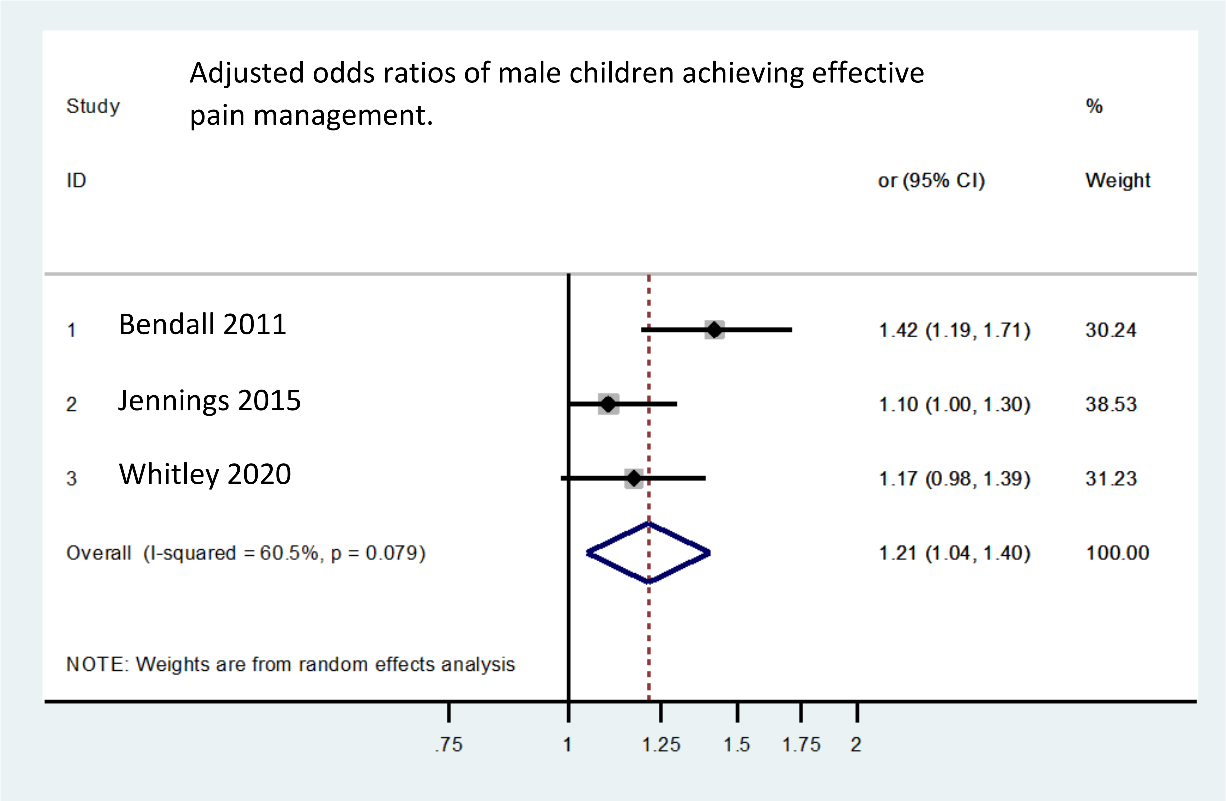

Heterogeneity chi-squared = 5.07 (d.f. = 2) p = 0.079

I-squared (variation in ES attributable to heterogeneity) = 60.5%

Estimate of between-study variance Tau-squared = 0.0104

Test of ES=1 : z= 2.54 p = 0.011

Predictive analgesics

Predictors (Analgesic)	Study			
	**Babl 2006*** [8] (administration to 10 minutes)	**Johansson 2013*** [9] (administration to final score)	**Karlsen 2014**† [3] (administration to final score)	**Murphy 2017**† [5] (administration to 10 minutes)
INF alone				5 (88%‡)
INF with other analgesic			4 (87%‡)	4 (79%‡)
Methoxyflurane with other analgesic	4.7			
Nasal s-ketamine with other analgesic		6.9 (100%‡)		

INF: Intranasal Fentanyl

*mean pain score reduction out of 11

†median pain score reduction out of 11

‡Percentage of patients achieving clinically meaningful reduction in pain (≥2 out of 11)

Thematic synthesis

Quote number	Quotes [source]	Initial codes	Descriptive themes	Analytical themes
1	"I had a sick 15-year old and one of the issues I had, he was given IV morphine after a musculoskeletal injury, but he came in the ambulance with his coach, it was a football match, and I had a lot of questions in my head about consent, you know? I suppose I erred on the side of doing the best for him I could or what I thought was the best for him at the time…" [Murphy and Barrett *et al* [14] pg496]	Concern about consent when administering analgesics	Education and training is considered poor by the majority of clinicians	**Internal Influences on the Clinician**
2	"As for education and guidelines, of course we're not allowed to give sufficiently high doses, even according to paediatric experts. The first thing they do at the receiving unit where we drop the child off is to supplement our pain treatment and that doesn't feel at all satisfactory." [Gunnvall and Augustsson *et al* [13] pg42]	Restrictive clinical guidelines inhibit effective pain management		
3	"I think from the training point of view, its two or three days in the paediatric A&E, in comparison to over two weeks in an adult A&E, with much more actual interaction with the staff and obviously clinical practice in terms of interventions…" [Murphy and Barrett *et al* [14] pg495]	Lack of exposure to children during education and training		
4	"When you are on placements, they are so precious about the children, you are not allowed near them for fear that you would upset them or make it worse…" [Murphy and Barrett *et al* [14] pg495]			
5	"Not much pediatric education in paramedic or EMT programs at any level of prehospital training . . . I don't think there's a lot of emphasis on pediatrics per se. In class we had I think five or six sessions on pediatrics and that's going through the whole gamut of everything that has to deal with pediatrics . . .. Pain management wasn't really covered that much at all." [Williams and Rindal *et al* [15] pg523]			
6	"[We] are not allowed to touch [pediatric patients] when you're in paramedic school so when you get out of paramedic school you're in a trend." [Williams and Rindal *et al* [15] pg523]			
7	"I don't know if you have all been involved in some of the elearning that PHECC have been doing and it's excellent…" [Murphy and Barrett *et al* [14] pg495]	e-learning is beneficial		
8	"eLearning …they're economical for their service provider, it wouldn't cost them money, and they're easy to do for people, you can do them in your own time, and for people who don't necessarily like attending formal courses and exams, there's less pressure, it's a route that's working well in other areas that I think might be of benefit..." [Murphy and Barrett *et al* [14] pg495]			
9	"I just felt if I missed the IV now, I'm after wasting five minutes missing an IV and that's five minutes closer to the hospital, so, having used intranasal midazolam a number of times, it's super, getting it out and drawing it up and giving it…you wouldn't even have your line and Tegaderm out by the time you have the intranasal midazolam given…" [Murphy and Barrett *et al* [14] pg496]	Preference to defer analgesic administration until hospital arrival	Clinicians fear treating children in pain	
10	"I'm not going to stay on scene for an extra ten minutes to insert a line and give morphine if the hospital is only 5			

	minutes down the road…” [Murphy and Barrett *et al* [14] pg496]			
11	“When we went through class we were always told to look for reasons to not give medication and there’s never a great reason to give morphine . . .. I don’t think we covered too much about it in class at all. I just remember the overall generalization of medications: always look for reasons not to give it.” [Williams and Rindal *et al* [15] pg523]			
12	"I deferred when close to the hospital because I think there’s more of a comfort level in the hospital. They deal with it more. I think they’re better. They have the ability to assess pain better than we do. They do drug dosages, which isn’t that big of a deal but it’s just something that they’re more comfortable with . . . .” [Williams and Rindal *et al* [15] pg524]			
13	“I mean all of the controlled substance charts are 100% QA’d, which I’m sure Dr. [agency medical director] reads as well . . . I know that our ALS chief reads it. So maybe that’s a part of it as far as deferring . . . ‘Am I really comfortable doing this, and if I’m not and I screw up am I gonna lose my job? Am I gonna lose my card? Am I gonna get kicked back down to a basic level?”’ [Williams and Rindal *et al* [15] pg524]			
14	“I’m stingy with all my drugs.” [Williams and Rindal *et al* [15] pg524]			
15	“I am indifferent to distance from the hospital in terms of whether to give it or not. If it’s indicated, might as well get it to them sooner than later.” [Williams and Rindal *et al* [15] pg524]			
16	“If I’m two minutes away from the hospital, it’s gonna take me longer to stop, start the IV, put the person on the monitor, put the pulse oximetry on cuz you gotta check for that respiratory effort, and then actually administer the medicine versus driving two and a half or three minutes and havin’ the hospital do it.” [Williams and Rindal *et al* [15] pg524]			
17	“Morphine is risky if you don’t know a child’s gonna have an allergic reaction to it.” [Williams and Rindal *et al* [15] pg523]	Concern for adverse effect when using strong analgesics		
18	“ . . .you know if you give an adult too much morphine for example and you make them hypotensive and you depress their respiratory rate and effort, you can fix that pretty quickly in an adult, but the repercussions of doing that in a little kid? The risk is higher.” [Williams and Rindal *et al* [15] pg523]			
19	“It can happen and then you overdose them based on that guesstimate [of the patient’s weight] for some [expletive] little pain problem? No, it’s not gonna fly. But if it’s something serious, like a femur fracture . . .then at least the ends justify the means. I can’t justify it for some [expletive].” [Williams and Rindal *et al* [15] pg523]			
20	“You do not have the same routine to take care of children, you do not meet children seven days a week, like adults ... but children are not like little adults anyway, they are something else that requires extra supervision of the doses .. and other things and that is a stress factor.. “ [Holmström and Junehag *et al* [12] pg24]			
21	“When we went through class we were always told to look for reasons to not give medication and there’s never a great reason to give morphine . . .. I don’t think we covered too much about it in class at all. I just remember the overall generalization of medications: always look for reasons not to give it.” [Williams and Rindal *et al* [15] pg523]			
22	“A child with a deformed arm is more likely to get significant analgesia than a child in severe abdominal pain, let’s say, and appendicitis…” [Murphy and Barrett *et al* [14] pg496]	Decision making; trauma is treated more readily than medical pain	Prior clinical experience influences pain management	
23	“People won’t even consider paracetamol or ibuprofen for tummy pain…” [Murphy and Barrett *et al* [14] pg496]			
24	“…We have a lot of barriers to IV access in younger children. The older ones wouldn’t be a major problem but certainly younger children, which again certainly affects your mind set in relation to using the likes of morphine…” [Murphy and Barrett *et al* [14] pg496]	Lack of confidence with IV analgesics		

25	"I find it really hard to judge when is the right time, when is someone bad enough to warrant inflicting more pain with a cannula, and then the possibility that you might stick it into them two or three times before you would get anywhere, I would say, and with 90% of kids, I would really have no cannula…" [Murphy and Barrett *et al* [14] pg496]			
26	"... Nowadays we don't always have to hurt the child by inserting a PVC ... since we have the intranasal technique. And then it could be so anyway, that I have to insert this ... It hurts and can be messy ... They are chubby at a certain age ... it is often difficult to find the vessels..." [Holmström and Junehag *et al* [12] pg26]			
27	"I think it's more of a familiarity and comfort issue. It's just not done often enough so that people are comfortable with it and will go ahead and utilize it . . .. People are just generally speaking afraid of kids because of a lack of familiarity and particularly pain management runs high on that list because it's one of the things we do least often." [Williams and Rindal *et al* [15] pg523]			
28	"I'm not that keen on treating pain in a child … because children incapable of communicating make me feel insecure, I don't know what effect my treatment is having. Is it bad, is it good, what information am I getting?" [Gunnvall and Augustsson *et al* [13] pg42]	Lack of prior clinical experience		
29	"When it comes to a paediatric emergency or an obstetric emergency, and it's just the exposure, we're not doing five of them a day, so I think we have to try and make up for that deficit somehow again be it in placements, be it in simulation…" [Murphy and Barrett *et al* [14] pg495]			
30	..."I must really try to gather my thoughts and have a mental preparation for how I should work directly in a place when arriving ..I have to show that this sort of thing is what I do every day; I am competent and it will be all right, I will take care of you." [Holmström and Junehag *et al* [12] pg25]			
31	"When it comes to children, we don't take histories, we don't actually have any hands-on experience and so our experience is very low. I think we are even at the stage whereby I think routinely we don't strip a child, we don't get them down to their nappy, we don't do that…" [Murphy and Barrett *et al* [14] pg496]			
32	"I knew he was in pain because of his presentation. He was screaming with any movement or palpation to the area. He was tachycardic too. His vital signs coincided with his presentation and his discomfort. I looked for elevated heart rate, elevated blood pressures." [Williams and Rindal *et al* [15] pg523]	Prior experience of managing pain is helpful		
33	"I can say I have to prepare myself during a trip to a severely ill child... because first of all, I have a noticeably higher rate of stress ... depending on the nature of the alarm, of course ...if it's a prior one and a bad case with a child involved, so to speak, then it is stressful" [Holmström and Junehag *et al* [12]pg25]	Raised clinician anxiety results in increased cautiousness		
34	"Makes you a little more anxious when you're dealing with a child. I feel that when our anxiety level is raised we're gonna be a little more hesitant about doing things that we should. A little more cautious I should say. Maybe it hinders our ability to assess the patient appropriately." [Williams and Rindal *et al* [15] pg524]			
35	"I have had a couple of appendicitis', I was at the GP's, and you go in there and the child is obviously in distress, in a lot of abdominal pain, and you're saying (to the GP), "Are you going to give him something for the pain?" And he's like, "No, you can't give him anything for the pain, it will only mask the symptoms when they get up to the hospital." So where do you go with that?" [Murphy and Barrett *et al* [14] pg496]	Discordance between HCPs is challenging	Colleagues influence the pain management process	**External Influences on the Clinician**
36	"It's very hard to turn around and say to parents, "I know the GP has said not to give analgesia but the ambulance driver is now saying, Oh I'm going to give them analgesia"… those are becoming issues as well..." [Murphy and Barrett *et al* [14] pg496]			
37	"It's something that could be in the back of your mind as well, the interaction you are having with the emergency			

	department staff when you get there, and you know that if this, if you are going to do something it's actually going to cause a difficulty even though it's within your scope. It may be something that contributes to your decision of whether or not to do it..." [Murphy and Barrett *et al* [14] pg496]			
38	"I think I may be more inclined to call for help from specialised units and the helicopter and such, as compared to when it's an adult." "Seek assistance from the resources at hand. We have good resources, we have specialised units and units with doctors in them and doctors on the phone." [Gunnvall and Augustsson *et al* [13] pg42]	Collaboration between HCPs can be helpful		
39	"On the best of days, we are two ambulances when there is a child involved ... then we are four people, which makes an opportunity to designate one person to take care of hysterical parents ... " [Holmström and Junehag *et al* [12] pg25]			
40	"Oh no, this child is reacting strongly against me somehow, you know. My voice or whatever, they can get scared. Then it might be better for the colleague to step in, much better." [Gunnvall and Augustsson *et al* [13] pg42]			
41	"Calling medical control at certain places around here and getting orders for pain control is an almost impossible task . . .. I have never successfully argued for a pain control order out of [hospital]. I have never successfully argued for a pain control order out of [hospital] for kids." [Williams and Rindal *et al* [15] pg523]	Clinical support is not beneficial		
42	"I think I may be more inclined to call for help from specialised units and the helicopter and such, as compared to when it's an adult." "Seek assistance from the resources at hand. We have good resources, we have specialised units and units with doctors in them and doctors on the phone." [Gunnvall and Augustsson *et al* [13] pg42]	Clinical support is beneficial		
43	"I feel that pediatric medical control doctors are more willing to work with you . . . having medical control doctors that are willing to chat with you on the phone definitely helps as far as increasing the usage of pain medication in the field." [Williams and Rindal *et al* [15] pg523]			
44	"When we got there [to the ED] I told them I gave 10 mg morphine and they flipped out. 'You gave 10 mg morphine?! Why'd you give 10 mg morphine?!' The doctor was cool with it. It was the nurses who were all flippin' out . . .. So that's another thing to keep in the back of my head. Am I gonna get yelled at by the hospital staff whether it's warranted or not?" [Williams and Rindal *et al* [15] pg523]	Negative judgement of colleagues hinders analgesic use		
45	"You know, we are not on a level footing, in terms of professionalism… Sometimes it's a mind-set in a particular department..." [Murphy and Barrett *et al* [14] pg496]			
46	"Depending on your boss of the year, some of them are in support of it, while some of them could care less. Our last boss used to brag about how we had the least narcotics administrations out of all the area paramedics." [Williams and Rindal *et al* [15] pg523]	Positive judgement of colleagues encourages analgesic use		
47	" . . .he [paramedic mentor] is very liberal with his pain meds . . . some of the paramedics that I've been trying to emulate are more liberal with their pain meds and I think that's what pushed me in that direction." [Williams and Rindal *et al* [15] pg523]	Confident mentors encourage analgesic administration		
48	"Well, I think that when we have children as patients, we often have several patients; even if we don't treat the adults, they play a big part in our handling of this instance of care." [Gunnvall and Augustsson *et al* [13] pg42]	Parents help the pain management process	Relatives on scene influence the pain management process	
49	"Talk to the parent first, take that detour, and try to keep the parent calm because how the parents are is reflected so much in the children, it's reflected a whole lot in the child." [Gunnvall and Augustsson *et al* [13] pg42]			
50	" . . .carry a Broselow tape and whip it out on every kid because I will admit that I struggle when it comes to judging a kid's weight . . .. If the parent knows and they're pretty reliable based on a well-baby checkup then I defer to the parent." [Williams and Rindal *et al* [15] pg523]			
51	"I have to establish contact so I can get close to the child; you have to learn to meet at their level. First of all, I			

	learned to kneel or on the floor so that we reach the same eye level. I've learned to ask questions so that the child understands me. Also, I've learned to meet the child and show that I'm a kind person and not a threat. How I do it depends a bit on what kind of child I have in front of me. If I have a child who does not even want to look at me, I may start with talking to Mom and Dad. " [Holmström and Junehag *et al* [12] pg25]			
52	"He measured my bloodoxygen (saturation)… Then he explained that it was really good, and then my son easily cooperated with the assessment…" [Jepsen and Rooth *et al* [11] pg5]			
53	"I would say it's 50% of the time they're helping, 50% of the time impeding, because you get the parents that are very supportive of what you're doing and they just kind of stand back and then you have the other parents that are in your face . . .." [Williams and Rindal *et al* [15] pg523]			
54	"I would say it's 50% of the time they're helping, 50% of the time impeding, because you get the parents that are very supportive of what you're doing and they just kind of stand back and then you have the other parents that are in your face . . .." [Williams and Rindal *et al* [15] pg523]	Parents hinder the pain management process		
55	"On the best of days, we are two ambulances when there is a child involved ... then we are four people, which makes an opportunity to designate one person to take care of hysterical parents ... " [Holmström and Junehag *et al* [12] pg25]			
56	"I've never had a parent get in the way as far as tellin' us how to treat, but I think maybe when they're upset because their child's hurt it does hinder our ability to take care of the patient in the way we're supposed to." [Williams and Rindal *et al* [15] pg523]			
57	"It's very important to alleviate children's pain. Especially thinking about their future healthcare, since they'll remember the second we get there until the second it no longer hurts. If we can make the pain disappear right away, then we've come a long way, then we're the heroes of the day." [Gunnvall and Augustsson *et al* [13] pg41]	Pain relief is important for the holistic care of the child	Child experience of event is important	**Child Factors**
58	"And I view this taking care of a child's pain, that it's not only a matter of taking care of the child but the whole situation around it, because it's the child's lifeworld I'm taking care of." [Gunnvall and Augustsson *et al* [13] pg42]			
59	"Its purpose is to lessen pain and to make things better for the patient and that's why we're here—to make the patient better." [Williams and Rindal *et al* [15] pg523]			
60	"Yes, I agree, but spontaneously, I would say that the primary focus is always the child. Parents will be secondary ... So, parents fall a little bit away. You get some kind of tunnel vision if there are few nurses in a place. It's the child and nothing else just then... until the child is stable ... then you can take care of the parents." [Holmström and Junehag *et al* [12] pg25]	Child's experience more important than parent's experience		
61	"… I usually prefer to do as much as possible in their home. Like we said before, then you can involve parents, colleagues, other relatives. And you can also involve the room, toys and such …." [Gunnvall and Augustsson *et al* [13] pg41]	Preference to treat at home in the child's own environment		
62	"But everything I'm going to do I explain first, and then, well, see the reaction. I want the child to participate, at least to have the sense of being in on it and making decisions." [Gunnvall and Augustsson *et al* [13] pg42]	Preference to involve the child in the clinical decision making		
63	"You know, I have to build up a relationship. Even if things happen quickly sometimes, I just must get the child to feel some kind of trust towards me, or it will be impossible for me to do anything at all. If not, I'll get nowhere in caring for the child, I won't even be able to alleviate the child's pain." [Gunnvall and Augustsson *et al* [13] pg41]	Developing trust between clinician and child is important		
64	..."I must really try to gather my thoughts and have a mental preparation for how I should work directly in a place when arriving ..I have to show that this sort of thing is what I do every day; I am competent and it will be all right, I			

	will take care of you." [Holmström and Junehag *et al* [12] pg25]			
65	"I have to establish contact so I can get close to the child; you have to learn to meet at their level. First of all, I learned to kneel or on the floor so that we reach the same eye level. I've learned to ask questions so that the child understands me. Also, I've learned to meet the child and show that I'm a kind person and not a threat. How I do it depends a bit on what kind of child I have in front of me. If I have a child who does not even want to look at me, I may start with talking to Mom and Dad. " [Holmström and Junehag *et al* [12] pg25]			
66	"… They played at the same time as they were assessing and giving him the treatment…" [Jepsen and Rooth *et al* [11] pg5]			
67	"I know my ambulance. I feel good, I like it there. I think I can convey this to the child: you'll like it here too." [Gunnvall and Augustsson *et al* [13] pg41]			
68	"I find it really hard to judge when is the right time, when is someone bad enough to warrant inflicting more pain with a cannula, and then the possibility that you might stick it into them two or three times before you would get anywhere, I would say, and with 90% of kids, I would really have no cannula…" [Murphy and Barrett *et al* [14] pg496]	Risk versus benefit of IV access		
69	"IVs are something we definitely don't like to do in kids. We cause them more pain starting IVs a lot of times . . .. Really don't like to do it . . .. That might be part of our decision as to whether or not we give pain management." [Williams and Rindal *et al* [15] pg523]			
70	"... Nowadays we don't always have to hurt the child by inserting a PVC ... since we have the intranasal technique. And then it could be so anyway, that I have to insert this ... It hurts and can be messy ... They are chubby at a certain age ... it is often difficult to find the vessels..." [Holmström and Junehag *et al* [12] pg26]			
71	"…We have a lot of barriers to IV access in younger children. The older ones wouldn't be a major problem but certainly younger children, which again certainly affects your mind set in relation to using the likes of morphine…" [Murphy and Barrett *et al* [14] pg496]			
72	"Not only did it relieve some of his pain, but it relieved some of his anxiety. Calmed him down a little bit more. It was easier to deal with him so it does have its benefits." [Williams and Rindal *et al* [15] pg523]	Analgesia improves child anxiety and compliance	Analgesics are helpful but administration is challenging	
73	"... Nowadays we don't always have to hurt the child by inserting a PVC ... since we have the intranasal technique. And then it could be so anyway, that I have to insert this ... It hurts and can be messy ... They are chubby at a certain age ... it is often difficult to find the vessels..." [Holmström and Junehag *et al* [12] pg26]	IV access is difficult, especially in younger children		
74	"If I've got a distressed toddler with a deformed upper limb...pain score of 10/10 (indicating severe pain). This child, like most, won't tolerate oral medication, is even less likely to cooperate with the administration of inhaled nitrous oxide. Securing vascular access is often technically challenging in children, for most APs, even for those experienced in cannulation, so even attempting the procedure will add to the child's anxiety and fear. So there's nothing we currently have that'll work, from a practical perspective. Clearly the intranasal route, if available, would prove ideal in this scenario." [Murphy and Barrett *et al* [14] pg497]			
75	"…We have a lot of barriers to IV access in younger children. The older ones wouldn't be a major problem but certainly younger children, which again certainly affects your mind set in relation to using the likes of morphine…" [Murphy and Barrett *et al* [14] pg496]			
76	"If you have a child that is vomiting and that you can't get a line on, you're kind of snookered as well because it eliminates everything you can do really, which is where your intranasal drug would come in fantastic..." [Murphy	Intranasal drugs may be beneficial when IV		

	and Barrett *et al* [14] pg496]	access is difficult		
77	"If I've got a distressed toddler with a deformed upper limb...pain score of 10/10 (indicating severe pain). This child, like most, won't tolerate oral medication, is even less likely to cooperate with the administration of inhaled nitrous oxide. Securing vascular access is often technically challenging in children, for most APs, even for those experienced in cannulation, so even attempting the procedure will add to the child's anxiety and fear. So there's nothing we currently have that'll work, from a practical perspective. Clearly the intranasal route, if available, would prove ideal in this scenario." [Murphy and Barrett *et al* [14] pg497]			
78	"... Nowadays we don't always have to hurt the child by inserting a PVC ... since we have the intranasal technique. And then it could be so anyway, that I have to insert this ... It hurts and can be messy ... They are chubby at a certain age ... it is often difficult to find the vessels..." [Holmström and Junehag *et al* [12] pg26]			
79	"I just felt if I missed the IV now, I'm after wasting five minutes missing an IV and that's five minutes closer to the hospital, so, having used intranasal midazolam a number of times, it's super, getting it out and drawing it up and giving it…you wouldn't even have your line and Tegaderm out by the time you have the intranasal midazolam given…" [Murphy and Barrett *et al* [14] pg496]			
80	" . . .carry a Broselow tape and whip it out on every kid because I will admit that I struggle when it comes to judging a kid's weight . . .. If the parent knows and they're pretty reliable based on a well-baby checkup then I defer to the parent." [Williams and Rindal *et al* [15] pg523]	Difficulty determining child's weight		
81	"I think that it is very effective (nitrous oxide) but I think you are limited by the fact that the patient is self-administering and has to understand kind of your instructions and so, you're kind of knocking out the younger paediatric age group straight away…" [Murphy and Barrett *et al* [14] pg496]	Inhaled analgesics are difficult to administer to younger children		
82	"…Your younger patients are effectively ruled out with the Entonox…" [Murphy and Barrett *et al* [14] pg496]			
83	"I am fully aware that a four-month-old baby will most likely not understand my reasoning, but maybe it can hear my voice and understand when I touch it." [Gunnvall and Augustsson *et al* [13] pg41]	Younger children are more difficult to assess	Assessment of children is challenging	
84	"How are you going to assess pain in children who cannot communicate, who are too small // Yeah, well, these preverbal children, it's very, very hard to communicate." [Gunnvall and Augustsson *et al* [13] pg42]			
85	"We don't actually perform assessments on very young children, so like say at the age of 3 and below, where almost you might as well take them out of the pain relief category because it's nearly impossible to assess it…" [Murphy and Barrett *et al* [14] pg495]			
86	"We're probably less equipped at the younger age and it's really just a general, your general impression…" [Murphy and Barrett *et al* [14] pg496]			
87	"Until they're actually at a stage where they can comprehend what you're saying or they can get to the stage where, they can understand the Wong–Baker chart, it's a bit hit-and-miss…" [Murphy and Barrett *et al* [14] pg496]			
88	"I think you hear how the little child screams and so on. You can recognise the type of scream. Whilst it gets more difficult, I think, when you get to teenagers and some older children. There can be a lot of difficult assessments with teenagers" [Holmström and Junehag *et al* [12] pg26]	Older children are more difficult to assess		
89	"Are you screaming because you're in pain? Are you screaming because you're sad? Are you screaming because you're afraid? Are you screaming because … well, I don't know." [Gunnvall and Augustsson *et al* [13] pg41]	Assessment of pain is very difficult in children		
90	"When you don't know why they are screaming, I think it's hard…" [Jepsen and Rooth *et al* [11] pg5]			
91	"When it comes to children, we don't take histories, we don't actually have any hands-on experience and so our experience is very low. I think we are even at the stage whereby I think routinely we don't strip a child, we don't get			

	them down to their nappy, we don't do that…" [Murphy and Barrett *et al* [14] pg496]			
92	"…I don't think it has taken the importance or it hasn't got to the same level of relevance as say, adult pain relief has, where that's a taken and it's a given that there will be pain relief given as early as possible…" [Murphy and Barrett *et al* [14] pg494]	Difference between treating adults and children is challenging		
93	"It's something I would look up just because it's not something that I do as often as other protocols. I would definitely need to look them [pediatric protocols] up more so than for adults . . .." [Williams and Rindal *et al* [15] pg523]			
94	"People aren't used to it and haven't gotten into the mind set that pain relief is an integral part of paediatric treatment…" [Murphy and Barrett *et al* [14] pg494]			
95	"You do not have the same routine to take care of children, you do not meet children seven days a week, like adults ... but children are not like little adults anyway, they are something else that requires extra supervision of the doses .. and other things and that is a stress factor.. " [Holmström and Junehag *et al* [12]]			
96	"Well, their play, in so far as … or, rather, kids' curiosity. All kids are curious. And that's also very important when, like, you see these tired, drooping, pain … if you see the slightest sign of curiosity in their eyes, then you know, well, it's not like … OK, the kid is sick, but not taking it so super seriously … A lot of times you get that feeling." [Gunnvall and Augustsson *et al* [13] pg42]	Physiological signs are helpful in identifying pain		
97	"I knew he was in pain because of his presentation. He was screaming with any movement or palpation to the area. He was tachycardic too. His vital signs coincided with his presentation and his discomfort. I looked for elevated heart rate, elevated blood pressures." [Williams and Rindal *et al* [15] pg523]			

IV – Intravenous, HCP – Health Care Professional

GRADE assessment

Identified predictor	Quality assessment					Summary of findings			
	Design	Quality	Consistency	Directness	Other modifying factors*	Number of patients	Effect	Quality**	Importance
							AORs (95% CI) [patient group (comparator)]		
Child gender (male)	Observational and other studies	No serious limitations	No important inconsistency	Some uncertainty about directness (people and outcome measure)	Sparse data	3312	1.42 (1.19–1.71) [males (compared to females)]	**Very Low**	Important
						15,016	1.1 (1.0-1.3) [males (compared to females)]		
						9833	1.27 (1.09-1.49) [males (compared to females)]		
						2312	1.17 (0.98-1.39) [males (compared to females)]		
Child age (younger)	Observational and other studies	No serious limitation	No important inconsistency	Some uncertainty about directness (people and outcome measure)	Sparse data	3312	1.33 (1.00–1.75) [5-9 years (compared to 10-15)]	**Very Low**	Important
						15,016	0.7 (0.6-0.95) [5-9 years (compared to 0-4)]		
						15,016	0.5 (0.4-0.6)		

							[10-14 years (compared to 0-4)]		
						9833	0.93 (0.41-2.10) [3-6 years (compared to <3 years)]		
						9833	0.60 (0.28-1.32) [7-9 years (compared to <3 years)]		
						9833	0.49 (0.23-1.06) [>9 years (compared to <3 years)]		
						2312	1.53 (1.18-1.97) [0-5 years (compared to 12-17 years)]		
						2312	1.49 (1.21-1.82) [6-11 years (compared to 12-17 years)]		
Type of pain (trauma)	Observational and other studies	No serious limitation	No important inconsistency	Some uncertainty about directness (people and outcome measure)	Sparse data	3312	0.69 (0.50-0.96) [Abdominal Pain/Problems (compared to trauma)]	**Very Low**	Important
						15,016	1.7 (1.5-1.9) [Musculoskeletal (compared to medical)]		
						15,016	1.6 (1.1-2.5) [Burns (compared to medical)]		
						15,016	1.4 (1.1-1.9) [Trauma (Other) (compared to medical)]		

						9833	0.45 (0.14-1.41) [Poisoning (compared to musculoskeletal)]		
						9833	0.22 (0.08-0.60) [Cardiac (compared to musculoskeletal)]		
						2312	1.18 (0.97-1.43) [Trauma (compared to medical)]		
Analgesic administration	Observational and other studies	No serious limitation	No important inconsistency	Some uncertainty about directness (people and outcome measure)	None	15,016	6.6 (5.9-7.3) [Any analgesic (compared to no analgesic)]	**Low**	Important
						2312	2.26 (1.87-2.73) [Analgesic administered (compared to no analgesic)]		
						268	Four studies demonstrated an association between analgesia administration and effective pain management		

AOR – Adjusted Odds Ratios

*Imprecise or sparse data, a strong or very strong association, high risk of reporting bias, evidence of a dose-response gradient, effect of plausible residual confounding.

****High** = Further research is very unlikely to change our confidence in the estimate of effect. **Moderate** = Further research is likely to have an important impact on our confidence in the estimate of effect and may change the estimate. **Low** = Further research is very likely to have an

important impact on our confidence in the estimate of effect and is likely to change the estimate. **Very low** = Any estimate of effect is very uncertain.

CERQual evidence profile

**Summary of review finding**	**Studies contributing to the review finding**	**Methodological limitations**	**Coherence**	**Adequacy**	**Relevance**	**CERQual assessment of confidence in the evidence**	**Explanation of CERQual assessment**
1. The ability of prehospital clinicians to effectively manage pain in children is influenced by internal factors such as fear, prior clinical experiences and education and training.	[12-15]	Minor concerns regarding methodological limitations that may reduce confidence in the review finding. (Two studies with no concern, one study with minor concern [insufficient rigorous data analysis] and one study with moderate concern [unclear justification for recruitment strategy, little reflexivity and insufficient rigorous data analysis])	No or very minor concerns about coherence	No or very minor concerns about adequacy	Minor concerns regarding relevance that may reduce confidence in the review finding. (All three studies represent three different sub-groups of EMS staff [paramedics, advanced paramedics and prehospital emergency nurses])	Moderate	Minor concerns regarding methodological limitations and relevance
2. The ability of prehospital clinicians to effectively manage pain in children is influenced by external factors such as colleagues	[11-15]	Minor concerns regarding methodological limitations that may reduce confidence in the review finding. (Three studies with no concern, one study with minor concern [insufficient rigorous data analysis] and one study with	No or very minor concerns about coherence	Moderate concerns about adequacy of data: all five studies offered limited thin	Minor concerns regarding relevance that may reduce confidence in the review finding. (All three studies represent three different sub-groups of	Low	Moderate concerns about adequacy of data and minor concerns about methodological limitations and relevance

and relative on scene.		moderate concern [unclear justification for recruitment strategy, no reflexivity and insufficient rigorous data analysis])		data, particularly around the influence of relatives on scene.	EMS staff [paramedics, advanced paramedics and prehospital emergency nurses])		
3. The ability of prehospital clinicians to effectively manage pain in children is influenced by child factors such as challenging pain assessment and analgesic administration and the perceived importance of the child's experience.	[11-15]	Minor concerns regarding methodological limitations that may reduce confidence in the review finding. (Three studies with no concern, one study with minor concern [insufficient rigorous data analysis] and one study with moderate concern [unclear justification for recruitment strategy, no reflexivity and insufficient rigorous data analysis])	No or very minor concerns about coherence	Minor concern about adequacy of data: Three studies offered limited data towards the 'importance of the child's experience' theme	Minor concerns regarding relevance that may reduce confidence in the review finding. (Four studies represent three different sub-groups of EMS staff [paramedics, advanced paramedics and prehospital emergency nurses])	Moderate	Minor concerns about methodological limitations, adequacy of data and relevance

CERQual summary of qualitative findings

Objective: To identify, appraise and synthesise qualitative research evidence on the barriers and facilitators to effective pain management in children by ambulance services<br>Perspective: Experiences and attitudes of clinicians, patients and relatives in any country			
Summary of review finding	Studies contributing to the review finding	CERQual assessment of confidence in the evidence	Explanation of CERQual assessment
1. The ability of prehospital clinicians to effectively manage pain in children is influenced by internal factors such as fear, prior clinical experiences and education and training.	[12-15]	Moderate	Minor concerns regarding methodological limitations and relevance
2. The ability of prehospital clinicians to effectively manage pain in children is influenced by external factors such as colleagues and relative on scene.	[11-15]	Low	Moderate concerns about adequacy of data and minor concerns about methodological limitations and relevance
3. The ability of prehospital clinicians to effectively manage pain in children is influenced by child factors such as challenging pain assessment and analgesic administration and the perceived importance of the child's experience.	[11-15]	Moderate	Minor concerns about methodological limitations, adequacy of data and relevance

References

[1] Bendall JC, Simpson PM, Middleton PM. Effectiveness of prehospital morphine, fentanyl, and methoxyflurane in pediatric patients. Prehospital Emergency Care. 2011;15(2):158-65. 10.3109/10903127.2010.541980.

[2] Jennings PA, Lord B, Smith K. Clinically meaningful reduction in pain severity in children treated by paramedics: a retrospective cohort study. The American journal of emergency medicine. 2015;33(11):1587-90. 10.1016/j.ajem.2015.06.026.

[3] Karlsen APH, Pedersen DMB, Trautner S, Dahl JB, Hansen MS. Safety of Intranasal Fentanyl in the Out-of-Hospital Setting: A Prospective Observational Study. Annals of Emergency Medicine. 2014;63(6):699-703. 10.1016/j.annemergmed.2013.10.025.

[4] Lord B, Jennings PA, Smith K. Effects of the Introduction of Intranasal Fentanyl on Reduction of Pain Severity Score in Children: An Interrupted Time-Series Analysis. Pediatric emergency care. 2019;35(11):749-54. 10.1097/pec.0000000000001376.

[5] Murphy AP, Hughes M, McCoy S, Crispino G, Wakai A, O'Sullivan R. Intranasal fentanyl for the prehospital management of acute pain in children. European Journal Of Emergency Medicine: Official Journal Of The European Society For Emergency Medicine. 2017;24(6):450-4. 10.1097/MEJ.0000000000000389.

[6] Whitley GA, Hemingway P, Law GR, Wilson C, Siriwardena AN. Predictors of effective management of acute pain in children within a UK ambulance service: A cross-sectional study. The American journal of emergency medicine. 2020;38(7):1534-40. 10.1016/j.ajem.2019.11.043.

[7] Downes MJ, Brennan ML, Williams HC, Dean RS. Development of a critical appraisal tool to assess the quality of cross-sectional studies (AXIS). BMJ Open. 2016;6(12):e011458. 10.1136/bmjopen-2016-011458.

[8] Babl FE, Jamison SR, Spicer M, Bernard S. Inhaled methoxyflurane as a prehospital analgesic in children. Emergency Medicine Australasia: EMA. 2006;18(4):404-10.

[9] Johansson J, Sjöberg J, Nordgren M, Sandström E, Sjöberg F, Zetterström H. Prehospital analgesia using nasal administration of S-ketamine - a case series. Scandinavian Journal of Trauma, Resuscitation and Emergency Medicine. 2013;21(1). 10.1186/1757-7241-21-38.

[10] Moola S MZ, Tufanaru C, Aromataris E, Sears K, Sfetcu R, Currie M, Qureshi R, Mattis P, Lisy K, Mu P-F. Chapter 7: Systematic reviews of etiology and risk. In: Aromataris E, Munn Z (Editors),. Joanna Briggs Institute Reviewer's Manual. The Joanna Briggs Institute.2017. Available from: https://reviewersmanual.joannabriggs.org/.

[11] Jepsen K, Rooth K, Lindstrom V. Parents' experiences of the caring encounter in the ambulance service - A qualitative study. Journal of clinical nursing. 2019. 10.1111/jocn.14964.

[12] Holmström MR, Junehag L, Velander S, Lundberg S, Ek B, Häggström M. Nurses' experiences of prehospital care encounters with children in pain. International emergency nursing. 2019;43:23-8. 10.1016/j.ienj.2018.07.004.

[13] Gunnvall K, Augustsson D, Lindström V, Vicente V. Specialist nurses' experiences when caring for preverbal children in pain in the prehospital context in Sweden. International emergency nursing. 2018;36:39-45. 10.1016/j.ienj.2017.09.006.

[14] Murphy A, Barrett M, Cronin J, McCoy S, Larkin P, Brenner M, et al. A qualitative study of the barriers to prehospital management of acute pain in children. Emergency Medicine Journal: EMJ. 2014;31(6):493-8. 10.1136/emermed-2012-202166.
[15] Williams DM, Rindal KE, Cushman JT, Shah MN. Barriers to and enablers for prehospital analgesia for pediatric patients. Prehospital Emergency Care: Official Journal Of The National Association Of EMS Physicians And The National Association Of State EMS Directors. 2012;16(4):519-26. 10.3109/10903127.2012.695436.
[16] Critical Appraisal Skills Programme. Critical Appraisal Skills Programme (CASP) Qualitative Research Checklist 2013 [Available from: http://docs.wixstatic.com/ugd/dded87_25658615020e427da194a325e7773d42.pdf.
